# Curcumin triggers reactive oxygen species-mediated apoptosis and suppresses tumor growth in metastatic oral squamous cell carcinoma

**DOI:** 10.3389/fonc.2025.1668271

**Published:** 2025-10-10

**Authors:** Tarsila de Carvalho Freitas Ramos, Rosane Borges Dias, Luciano de Souza Santos, Ludmila de Faro Valverde, Raphael Luís Rocha Nogueira, Iasmin Nogueira Bastos, Ricardo Della Coletta, Milena Botelho Pereira Soares, Bruno Solano de Freitas Souza, Jean Nunes dos Santos, Mitermayer Galvão dos Reis, Daniel Pereira Bezerra, Clarissa Araújo Gurgel

**Affiliations:** ^1^ Gonçalo Moniz Institute, Oswaldo Cruz Foundation (IGM-FIOCRUZ/BA), Salvador, Brazil; ^2^ Department of Propedeutics, School of Dentistry of the Federal University of Bahia, Salvador, Brazil; ^3^ Department of Health, School of Dentistry of the State University of Feira de Santana, Feira de Santana, Brazil; ^4^ Department of Biological Sciences, State University of Feira de Santana, Feira de Santana, Brazil; ^5^ Center for Biotechnology and Cell Therapy, D’Or Institute for Research and Education (IDOR), São Rafael Hospital, Salvador, Brazil; ^6^ Department of Dentistry, Federal University of Sergipe, Lagarto, Brazil; ^7^ Department of Pathology and Legal Medicine, School of Medicine of the Federal University of Bahia, Salvador, Brazil; ^8^ Department of Oral Diagnosis, School of Dentistry, University of Campinas, Piracicaba, Brazil; ^9^ Institute of Innovation in Advanced Health Systems (ISI SAS), University SENAI/CIMATEC, Salvador, Brazil

**Keywords:** oral cancer, cytotoxicity, natural compounds, 3D cell culture, xenograft model

## Abstract

**Background:**

Oral squamous cell carcinoma (OSCC) remains a major clinical challenge with limited effective treatment options. In this context, several natural compounds (NC), such as curcumin, have shown promising effects in OSCC. However, there is still limited evidence about curcumin’s effects on cell death in metastatic OSCC cells and its cytotoxicity in preclinical models. To address this gap, this study aimed to evaluate the effects of curcumin on mitochondrial stress–induced apoptotic cell death and its cytotoxicity in preclinical models.

**Methods:**

Curcumin’s cytotoxicity was assessed in both 2D (monolayer) and 3D (spheroid model) cell cultures using a luminescent assay. Additionally, morphological parameters (FSC and SSC), apoptosis, and reactive oxygen species (ROS) production were analyzed in 2D cell cultures by flow cytometry, while morphological changes were evaluated in 3D cultures through microscopy. The *in vivo* assay was performed using a xenograft model in mice (C.B-17 SCID).

**Results:**

Curcumin demonstrated cytotoxicity in 2D cell cultures, induced apoptosis, and increased ROS production, effects that were confirmed with antioxidant pretreatment (N-acetyl-L-cysteine). In the 3D cell culture, curcumin caused loss of spheroid integrity, suppressed tumor growth, and reduced tumor emboli and metastatic nodules in mice.

**Conclusion:**

Our findings suggest that curcumin induces cell death via apoptosis mediated by oxidative stress and exhibits promising cytotoxic activity in the spheroid model, while also inhibiting OSCC growth in mice.

## 1 Introduction

Oral cancer is a serious global health concern and the 13th most common type of tumor (1). Among oral cancers, oral squamous cell carcinoma (OSCC) accounts for up to 90% of all cases (1, 2). Despite improvements in treatment, the morbidity and mortality rates remain high, mainly because of late diagnosis, with a five-year survival rate of about 50% (3), significantly affecting patients’ quality of life (4). In addition, late diagnosis and drug resistance in tumor cells are major challenges in cancer treatment (2, 5–7).

Currently, basic, translational, and clinical research efforts focus on exploring tumor biology and developing new, effective therapies to address these issues. In this scenario, natural compounds (NC) are being investigated for their pharmacological potential in cancer treatment, as many chemotherapy drugs used clinically, such as paclitaxel, docetaxel, and vincristine, were derived from these substances (8–10). Indeed, NC represent an important approach for cancer therapy (10–14) and, in general, possible mechanisms of action include DNA damage, induction of apoptosis, cell cycle arrest, generation of reactive oxygen species (ROS), and the ability to stabilize and inactivate free radicals (8–10, 12). Consequently, demonstrating pharmacologically meaningful activity of NC requires proper controls and a clear correlation between extract activity and isolated pure compounds (15). Furthermore, it is essential to use test concentrations that are realistically achievable *in vivo*, avoiding effects observed only at artificially high doses (16).

In this effort, aiming to use an NC with fewer side effects, lower cost, and promising results in OSCC, curcumin [1,7-bis (4-hydroxy-3-methoxyphenyl) −1,6-heptadiene-3,5-dione)] stands out (10, 17). The *Curcuma* genus has a long history of medicinal applications, composed of approximately 120 species. Among the *Curcuma* species, *Curcuma longa L*. is the most widely recognized (18), and curcumin is a major constituent of turmeric (19). The pharmacological activity of turmeric is mainly attributed to curcuminoids consisting of curcumin and two related compounds, namely dimethoxy curcumin [4-hydroxycinnamoyl-(4-hydroxy-3-methoxyc innamoyl) methane] and bis-dimethoxy curcumin [bis-(4-hydroxycinnamoyl) methane], which exhibit varying degrees of antioxidant, anti-inflammatory, and anticancer activities (19–21).

Curcumin has a well-established safety record in both animals and humans, even at doses up to 8 g/day, and is recognized as GRAS (generally recognized as safe) by the FDA (18). Despite its well-established safety, some reports have highlighted mild side effects under certain conditions. In humans, doses of 0.45–12 g/day have been associated with gastrointestinal symptoms, headache, rash, and transient increases in liver enzymes (22). Similarly, patients receiving 1.5 g/day for 4 weeks reported minor effects such as constipation and stomachache, without major toxicity (23). Moreover, curcumin at 3.6 g/day for 6 months was well-tolerated in leukoplakia patients, with no severe adverse effects reported (24).On the other hand, the hydrophobic nature of curcumin after oral administration triggers a poor absorption rate by the gastrointestinal (GI) tract may limit its therapeutic use in clinical practice (18).

Regarding anticancer effects, several activities have been reported, including the suppression of cell proliferation, inhibition of angiogenesis, and induction of cell death in various malignancies, such as colorectal (25), breast (26), biliary (27), and prostate cancers (28). Moreover, extensive research has elucidated multiple molecular mechanisms through which curcumin exerts anticancer effects. Curcumin promotes apoptosis by upregulating pro-apoptotic proteins and downregulating anti-apoptotic proteins (29), elevates ROS levels via mitochondrial dysfunction and DNA damage (30–32), and induces G2/M phase arrest in various cancer cell lines (31–33). Additionally, curcumin downregulates the Wnt/β-catenin signaling pathway, which is involved in maintaining stemness and promoting proliferation in cancer cells (34), and inhibits enzymes associated with extracellular matrix degradation and tumor invasion (35).

Considering that the literature is still limited regarding the effects of curcumin in oral cancer (10, 17, 36, 37), this study hypothesizes that curcumin exerts promising cytotoxic activity against metastatic OSCC cells by reducing cell proliferation and inducing apoptosis through an oxidative stress–mediated mechanism, in addition, exhibit cytotoxic effects in the 3D cell culture (spheroids), which mimics several features of the tumor microenvironment, enabling evaluation of drug resistance. Thus, curcumin exhibits antitumor effects in a xenograft mice model, which offers greater complexity and translational relevance. Accordingly, this study aimed to evaluate the effects of curcumin on mitochondrial stress–induced apoptotic cell death in 2D cell culture and its cytotoxic effects in a 3D cell culture, as well as in a xenograft model of OSCC.

## 2 Materials and methods

### 2.1 Experimental study

**Figure 1:**
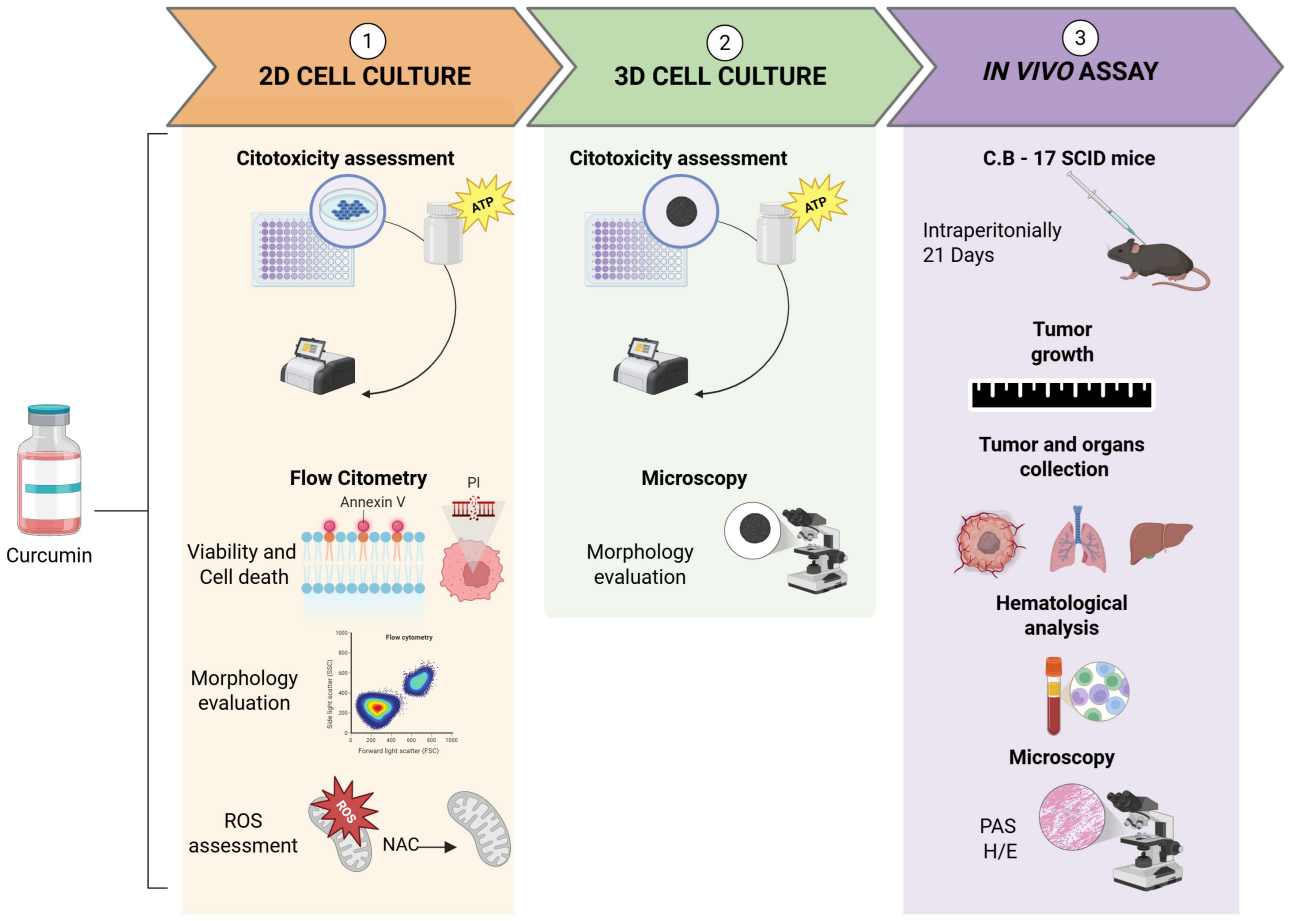
Flowchart depicting *in vitro* and *in vivo* experimental design. Created with BioRender.com.

The workflow summarized in **Figure 1** outlines the methods applied in this study. Briefly, after cell culture and maintenance (Section 2.3), cytotoxicity was assessed in 2D cell culture (Section 2.4), and flow cytometry was used to evaluate cell viability, death patterns, morphology (Section 2.5), and ROS production (Section 2.6). Next, 3D cell culture was applied (Section 2.7), followed by cytotoxicity assessment (Section 2.4) and morphology evaluation (Section 2.7). Finally, the *in vivo* assay was conducted in mice (Section 2.8), with tumor growth (Section 2.8), toxicological and hematological evaluation (Section 2.8.1) and histological analyses (Section 2.8.2).

### 2.2 Drug specifications

In this study, curcumin (C1386, Sigma-Aldrich, São Paulo, Brazil) and 5-fluorouracil (5-FU, Sigma-Aldrich, St. Louis, MO, USA) were individually weighed (5 mg), and dissolved in DMSO(C2H6OS, Dimethyl sulfoxide, Panreac) to prepare stock solutions (5 mg/mL) and then diluted to obtain working solutions at 1 mg/mL. All stock solution aliquots were stored at −80°C, while working solutions were kept at −20°C.

### 2.3 Cell culturing and maintenance

This study focused on the highly metastatic HSC3 cells (Human oral squamous carcinoma cell line). HSC3 cells (JCRB Cell Bank, Osaka, Japan) were placed in flasks (75 cm^3^, 250 mL volume) containing DMEM medium (Gibco, Life Technologies, Gaithersburg, MD, USA) supplemented with 10% fetal bovine serum (FBS, Gibco, Life Technologies, Gaithersburg, MD, USA), 1% penicillin, 1% streptomycin (Gibco, Life Technologies, Gaithersburg, MD, USA), and 0.8% hydrocortisone (Sigma-Aldrich, St Louis, MO, USA). Cells were cultured and kept in incubators, under an atmosphere of 5% CO_2_ at 37 °C. To monitor cell growth, an inverted microscope (EVOS™, Invitrogen™) was used daily and trypsin (0.5% Trypsin-EDTA) (Gibco, Life Technologies, Gaithersburg, MD, USA) was used to dissociate cells when cell growth reached the necessary confluence (70-80% of the total culture flask volume). The HSC3 cell line was tested periodically for mycoplasma using a luminometer, according to the MycoAlert™ PLUS Mycoplasma Detection Kit (Lonza Bioscience, Morrisville, NC, USA).

### 2.4 Cytotoxicity assessment (2D/3D models)

To determine the EC50 (50% effective concentration), cellular ATP levels were measured using luminescence (CellTiter-Glo^®^ kit, Promega, Madison, WI, USA). For 2D monolayer and 3D spheroid cultures, cells were seeded at 0.7 × 10^5^ cells/mL and 5 × 10³ cells/well in 96-well plates, respectively (see Section 2.8 for 3D culture details). Cells were exposed to compounds, medium, or vehicle controls for 24 hours. In monolayers, curcumin and 5-FU were tested in serial dilutions from 0.19 to 25 µg/mL, while in spheroids, curcumin was tested from 0.14 to 300 µg/mL. Following treatment, cells were transferred to opaque 96-well plates (SPL, 30396), and viability was assessed using CellTiter-Glo^®^ 2.0 (monolayers) or CellTiter-Glo^®^ 3D (spheroids) at a 1:1 ratio with the culture medium, following the manufacturer’s instructions. Plates were shaken for 5 minutes to mix, incubated at room temperature in the dark for 25 minutes, and luminescence was recorded using a multimode microplate reader (FilterMax F3, Molecular Devices) with SoftMax Pro software (v6.2.1). Three independent experiments were carried out, each in three replicates per experiment.

### 2.5 Evaluation of curcumin treatment on cell viability, pattern of death, and morphology in monolayer culture

To assess cell viability and death after 24 and 48 hours of treatment, HSC3 cells (0.7 × 10^5^ cells/2 mL in 6-well plates) were stained with annexin V-FITC and propidium iodide (PI) following the manufacturer’s instructions (BD Biosciences, Franklin Lakes, NJ, USA). After centrifugation, 100 µL of binding buffer containing 2 µL each of annexin V-FITC and PI was added, followed by a 15-minute incubation in the dark and addition of 100 µL binding buffer. Forward scatter (FSC) and side scatter (SSC) were analyzed using a BD LSRFortessa^®^ flow cytometer (FACSDiva v6.2), and FlowJo (v10, FlowJo LLC, Ashland, OR, USA) was used to quantify apoptotic cells and evaluate morphology. Cellular debris was excluded, and 10,000 events were collected per sample. Three independent experiments were carried out, each in two replicates per experiment.

### 2.6 Assessment of curcumin-induced pro-oxidant activity and reactive oxygen species production in monolayer culture

To evaluate the pro-oxidant effect of curcumin after 24 hours of treatment, HSC3 cells (0.7 × 10^5^ cells/2 mL) were seeded in 6-well plates. The fluorogenic probe MitoSOX™ (1 µM; Thermo Fisher Scientific, Waltham, MA, USA) was added to detect mitochondrial superoxide anion. To assess whether an antioxidant could block curcumin-induced cell death, cells were pretreated with 5 mM N-acetyl-L-cysteine (NAC; Sigma-Aldrich, St. Louis, MO, USA) for 1 hour before curcumin exposure. All samples were analyzed by flow cytometry as described in section 2.5. Three independent experiments were performed, each in two replicates.

### 2.7 3D cell culture

For spheroid formation, a protocol for homotypic OSCC spheroids was applied (38). Briefly, HSC3 cells were magnetized (Nanoshuttle-PL, Greiner Bio One) and, after successive centrifugations, were seeded (5 × 10^3^ cells/well) in a 96-well repellent plate (Ultra-Low Attachment Surface, Costar^®^). Next, a magnet (96 neodymium magnets, Nano3D Biosciences) was used to induce aggregation and print a spheroid at the bottom of each well. Spheroids were supplied and maintained as previously described in topic 2.3.

To evaluate the viability after 24 hours of treatment, the cellular ATP metabolism using luminescence (CellTiter-Glo^®^ 3D, Promega, Madison, Wisconsin, USA) was applied. The reagent was applied as previously described in section 2.4. Three independent experiments were carried out, each in two replicates per experiment.

### 2.8 Human OSCC xenograft model

The *in vivo* assessment was carried out as previously described (39). A total of 24 C.B-17 severe combined immunodeficient (SCID) mice (females, 25-30g) were obtained and kept at the animal facilities of the Gonçalo Moniz Institute-FIOCRUZ (Salvador, Bahia, Brazil). All animals were housed in cages with free access to food and water. The animal ethics committee of the Gonçalo Moniz Institute (CEUA, IGM, FIOCRUZ, Bahia) approved the experimental protocol used (number 001/2021).

Curcumin was dissolved in DMSO and diluted in distilled water (obeying the proportion of 5% DMSO), and the treatment was carried out intraperitoneally, once a day, for 21 days. The mice were divided into three groups: Group 1 (negative control group) - animals treated with 5% DMSO vehicle; Group 2 (positive control group) - animals treated with 5-FU (15 mg/kg); Group 3 - animals treated with Curcumin (50 mg/kg), and the treatments started 24 hours after inoculation. At the end of treatment, peripheral blood samples from the mice were collected for hematological analysis. The euthanasia of the animals was performed through an intraperitoneal injection with the anesthetic thiopental, and the tumors were removed and weighed, in addition to the liver, lung, heart, and kidneys of the mice. Treatment effects were expressed as the percentage inhibition of control.

#### 2.8.1 Toxicological and hematological evaluation

Mice were weighed at both the beginning and end of the experiment. Throughout the study, all animals were monitored for clinical signs of abnormality. The liver, kidneys, lungs, and heart were collected, weighed, and examined for lesions, discoloration, or hemorrhage. Hematological parameters were also evaluated, including total erythrocyte and leukocyte counts, as well as differential leukocyte counts (neutrophils, lymphocytes, and monocytes). Additionally, hemoglobin concentration and mean corpuscular volume were measured.

#### 2.8.2 Histological analysis

Tumors and organs were fixed in 10% buffered formalin and subsequently processed for histological analysis. Sections of 4 µm thickness were prepared from paraffin-embedded blocks and stained with hematoxylin and eosin (H&E). Histological evaluation was performed by an experienced pathologist using light microscopy (Olympus BX41) at magnifications of 4×, 100×, 200×, and 400× when necessary. Histological features were graded as negative (0), mild (+1), moderate (+2), or intense (+3). Tumor characteristics and histopathological changes were assessed through H&E staining, and histological grading was performed based on the World Health Organization Classification (WHO, 2022).

Liver sections were additionally stained with periodic acid–Schiff (PAS) to enhance detection of glycogen content and confirm the presence of hydropic degeneration. Histopathological changes in the heart, lungs, and kidneys were evaluated using H&E staining.

### 2.9 Statistical analyses

All results were compiled and analyzed statistically using GraphPad Prism software (version 8.4.2; GraphPad Software, Inc., San Diego, USA), based on data distribution assessed by the Gaussian curve. EC_50_ values were calculated by non-linear regression of the relative light units (RLU) using the formula: RLU (Drug)/RLU (Vehicle). Differences between groups were evaluated by analysis of variance (ANOVA), followed by the Student–Newman–Keuls *post hoc* test. Statistical significance was considered for p-values ≤ 0.05.

## 3 Results

### 3.1 Curcumin exerts cytotoxicity and reduces cell viability in the OSCC monolayer cell culture

**Figure 2:**
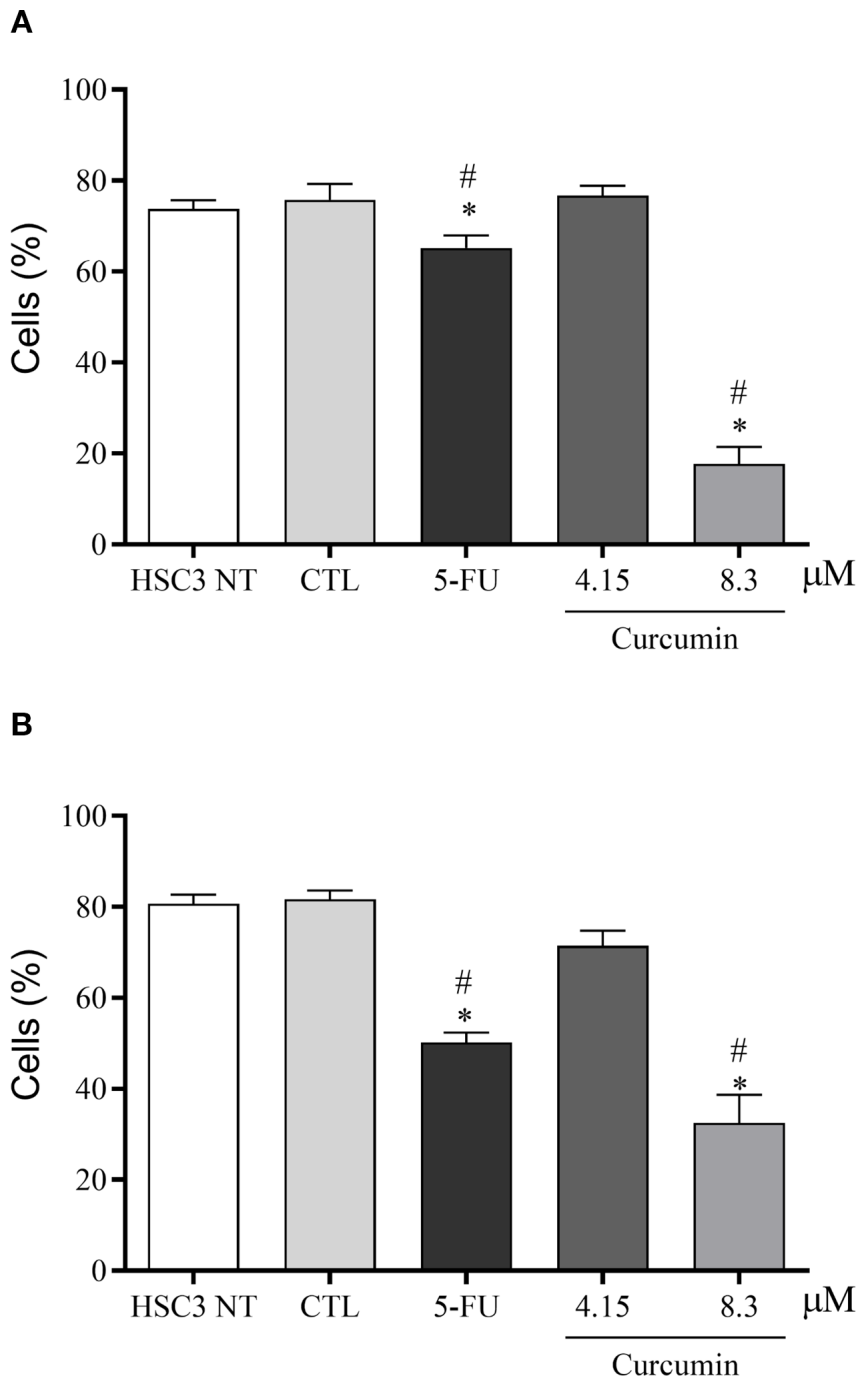
Analysis of cell viability through the Annexin V/Propidium iodide assay in HSC3 cells treated for 24 **(A)** and 48 **(B)** hours with Curcumin. Curcumin significantly decreased the viability of HSC3 cells after 24 hours of treatment **(A)** and after 48 hours of treatment **(B)**. The negative control was treated with the vehicle (DMSO) used to solubilize and dilute the substances, and 5-FU was used as a positive control. Data are representative of three independent experiments carried out, each in two replicates per experiment. Cellular debris was omitted from analysis, and 10,000 events were analyzed per sample. Viable cells were negative for the fluorogens Annexin V and Propidium iodide. (*) p ≤ 0.05 when compared to the negative control (DMSO) and (#) when compared to HSC3-NT (non-treated cells) by ANOVA (analysis of variance) followed by Student Newman-Keuls test.

In the 2D cell culture model, curcumin and 5-FU exhibited cytotoxicity in the HSC3 cell line, with EC50 values of 8.3 µM and 21.26 µM, respectively (**Supplementary Figure S1**). Additionally, curcumin significantly reduced the viability of HSC3 cells after 24 and 48 hours compared to the negative control (**Figure 2**).

### 3.2 Curcumin increases apoptosis and promotes changes in cell morphology

**Figure 3:**
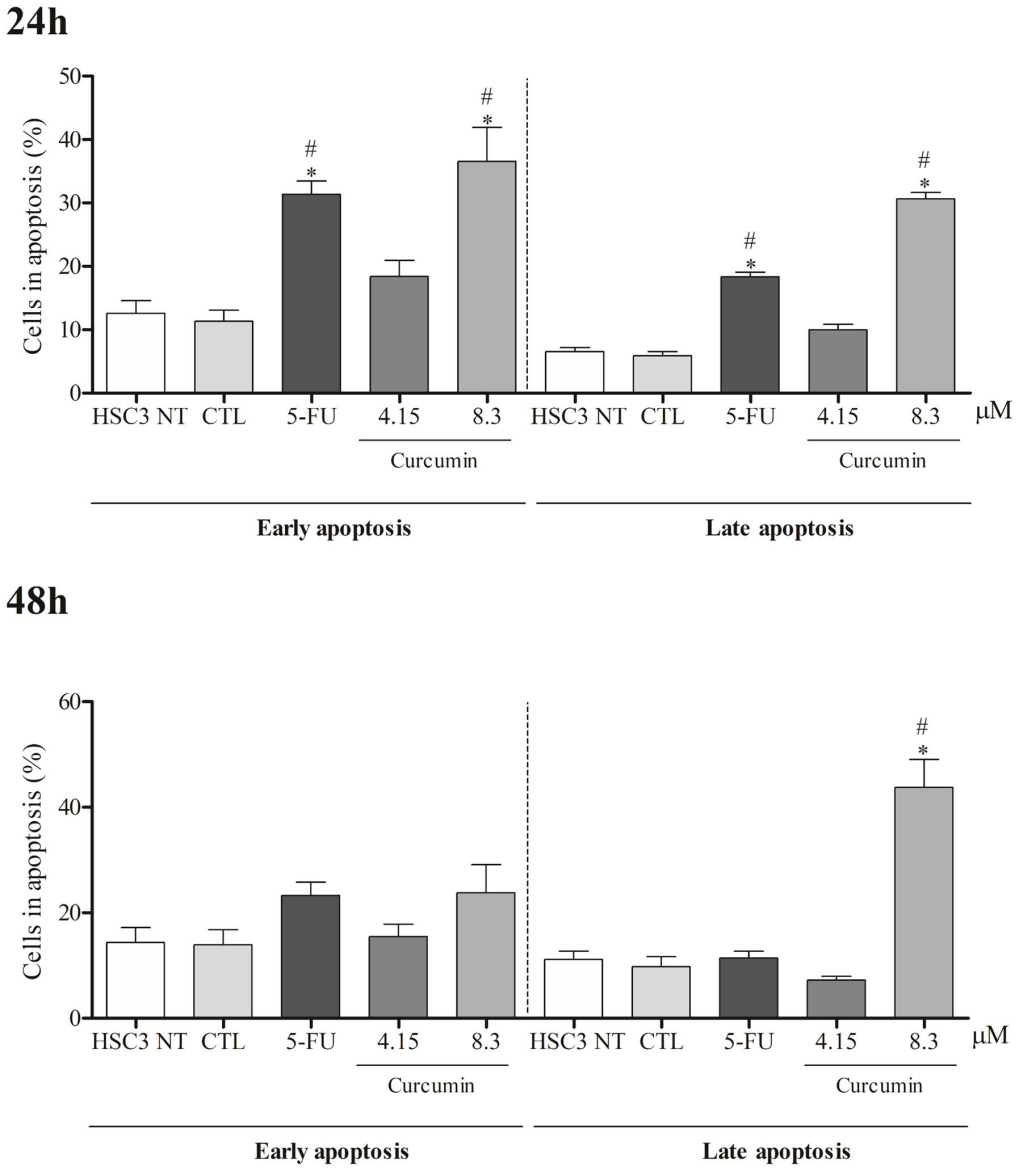
Analysis of apoptosis induced by Curcumin in HSC3 cells, after 24 and 48 hours of treatment. The negative control was treated with the vehicle (DMSO) used to solubilize and dilute the substances, and 5-FU was used as a positive control. Data are representative of three independent experiments carried out, each in two replicates per experiment. Cellular debris was omitted from analysis, and 10,000 events were analyzed per sample. (*) p ≤ 0.05 when compared to the negative control (DMSO) and (#) when compared to HSC3-NT (non-treated cells) by ANOVA (analysis of variance) followed by Student Newman-Keuls test.

After 24 hours, curcumin markedly increased late apoptosis in HSC3 cells, while at 48 hours, both curcumin and 5-FU significantly elevated early and late apoptosis (**Figure 3**). Notably, no rise in necrosis was observed under either treatment (**Supplementary Figure S2**). In parallel, curcumin induced cell shrinkage, reflected by reduced forward scatter (FSC), and nuclear condensation, evidenced by increased side scatter (SSC). Similarly, 5-FU, used as a positive control, triggered morphological alterations consistent with apoptosis. These effects proved to be both concentration- and time-dependent (**Supplementary Figure S3**).

### 3.3 Curcumin triggers ROS-mediated cell death in the OSCC, reduced by NAC pretreatment

**Figure 4:**
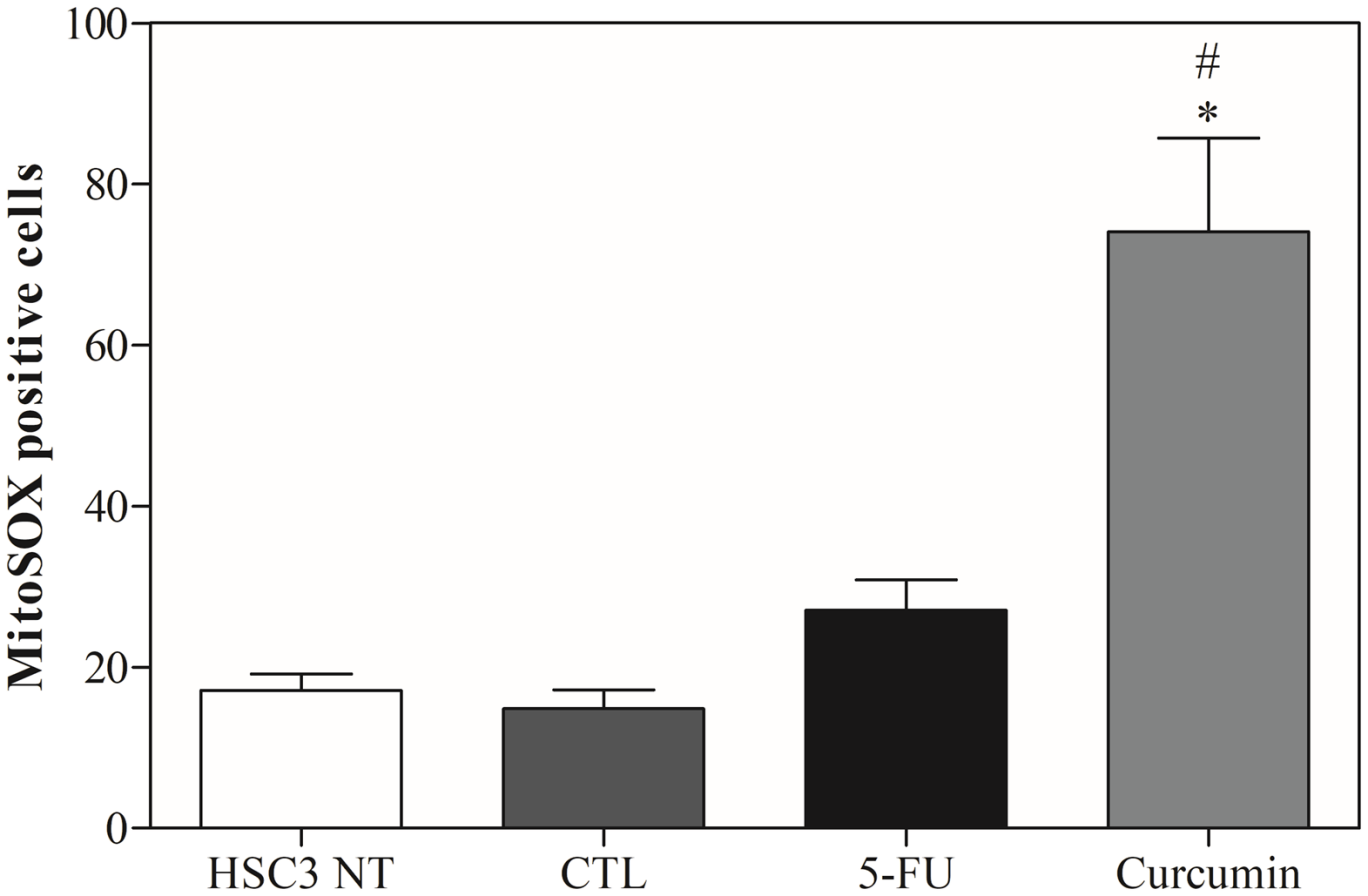
The effects of Curcumin on mitochondrial superoxide production in HSC3 cells, determined by the MitoSOX Assay, after 24 hours of treatment. The negative control was treated with the vehicle (DMSO) used to solubilize and dilute the substances, and 5-FU was used as a positive control. Values ​​respond to the mean ± S.E.M. of three independent experiments carried out, each in two replicates per experiment. Cellular debris was omitted from analysis, and 10,000 events were analyzed per sample. (*) p ≤ 0.05 when compared to the negative control (DMSO) and (#) when compared to HSC3-NT (non-treated cells) by ANOVA (anal ysis of variance) followed by Student Newman-Keuls test.

**Figure 5:**
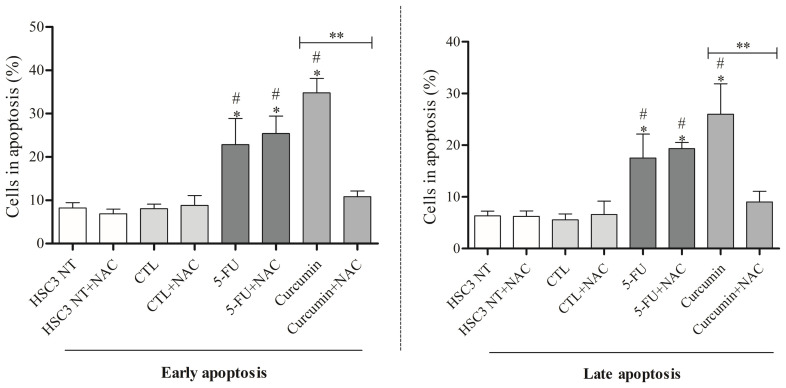
Effect of Curcumin (48 hours of treatment) on reactive oxygen species levels in HCS3 cells determined by flow cytometry with NAC pretreatment. The negative control was treated with the Vehicle (DMSO) used to solubilize and dilute the test substances. Cellular debris was omitted from analysis, and 10,000 events were analyzed per sample. Values respond to the mean ± S.E.M. of three independent experiments carried out, each in two replicates per experiment. (*) p ≤ 0.05 when compared to the negative control (DMSO) and (#) When compared to HSC3-NT (non-treated cells). (**) comparisons between Curcumin and Curcumin+NAC. Statistical analyses were performed using ANOVA (analysis of variance) followed by Student Newman-Keuls test.

Curcumin promoted a significant increase in mitochondrial superoxide in HSC3 cells (**Figure 4**). To confirm ROS involvement, cells were pretreated with the antioxidant N-acetylcysteine (NAC). NAC attenuated curcumin-induced ROS generation, supporting that its cytotoxic effects are mediated, at least in part, by oxidative stress, as shown in **Figure 5**.

### 3.4 Curcumin exerts cytotoxicity and reduces the viability in the OSCC 3D cell culture

**Figure 6:**
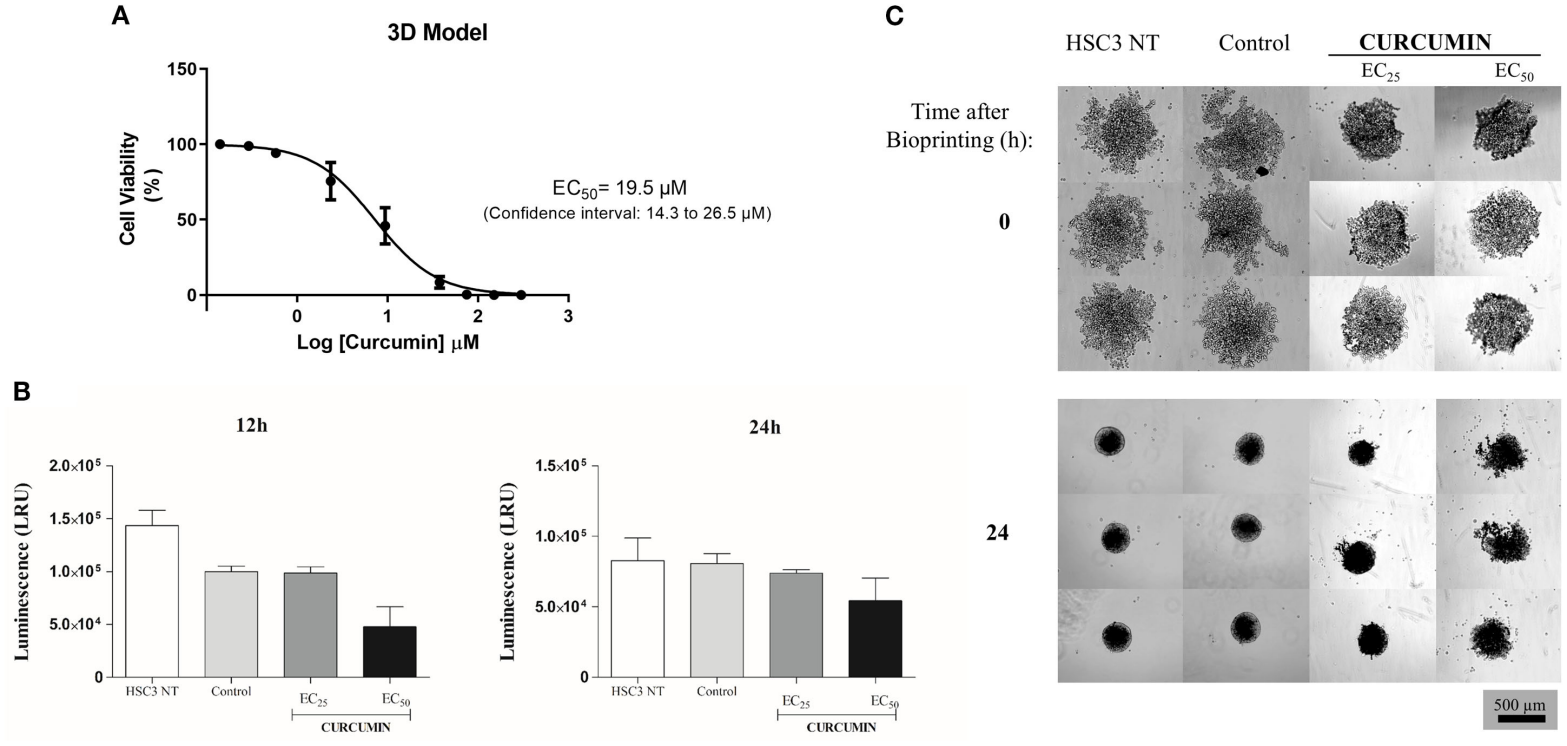
**(A)**The EC_50_ graded dose-response curves for Curcumin after 24h of treatment in a three-dimensional model. Data represented with EC_50_ values ​​in µM with a 95% confidence interval obtained by non-linear regression of three independent experiments carried out, each in three replicates per experiment, using the reagent CellTiter-Glo^®^ 3D. Values of luminescence (RLU) were calibrated using the negative control (DMSO). **(B)** Cytotoxic effect of Curcumin in OSCC 3D model after 12 and 24 hours of treatment. Values ​​respond to the mean ± S.E.M. of three independent experiments carried out in duplicate. (*) p ≤ 0.05 when compared to the negative control (DMSO) by ANOVA (analysis of variance) followed by Student Newman-Keuls test. **(C)** Morphological aspects of the spheroids after 24 hours of treatment with Curcumin. Spheroids treated with curcumin exhibited cellular disaggregation, disorganization of the spheroidal structure, and destruction of the outermost layers of the proliferative zone (EVOS,Thermo Fisher Scientific, 20x).

A homotypic spheroid formation assay with HSC3 cells was used to assess curcumin’s cytotoxicity. Curcumin reduced cell viability after 12 and 24 hours of exposure, with an EC_50_ of 19.5 µM at 24 hours (**Figures 6A, B**). Morphological analysis revealed marked structural disruption, including cell disaggregation, loss of spheroid organization, and destruction of the outer proliferative layers (**Figure 6C**), supporting its cytotoxic activity in 3D culture.

### 3.5 Curcumin suppresses tumor growth in a xenotransplant model of OSCC

**Figure 7:**
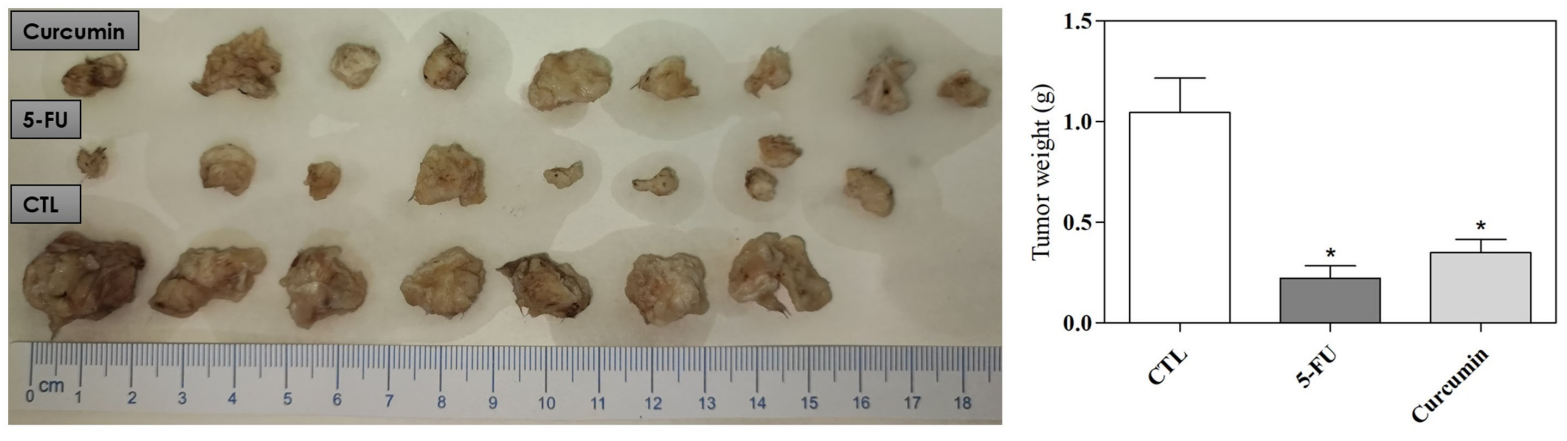
*In vivo* antitumor activity of Curcumin in C.B-17 SCID mice with HSC3 cell xenografts. Quantification of tumor weight and tumor inhibition. A heterotopic xenograft model was employed to evaluate *in vivo* antitumor effects, with 24 mice receiving subcutaneous injections of HSC-3 cells (1 × 10^7^ cells/500 μL) in the left axilla. The negative control (CTL) was treated with the vehicle (5% DMSO) used for diluting the compounds tested, and 5-fluorouracil was used as a positive control. Data are presented as themeans ± S.E.M. of 7–9 animals. (*) p ≤ 0.05 compared with the negative control by ANOVA, followed by the Student–Newma–Keuls test.

The CB17 SCID mice were inoculated with HSC3 cells. 24 hours later, treatment was initiated for 21 consecutive days. The animals were treated with curcumin at a dose of 50 mg/kg. **Figure 7** shows that Curcumin promoted a significant reduction in tumor growth compared to the negative control.

**Figure 8:**
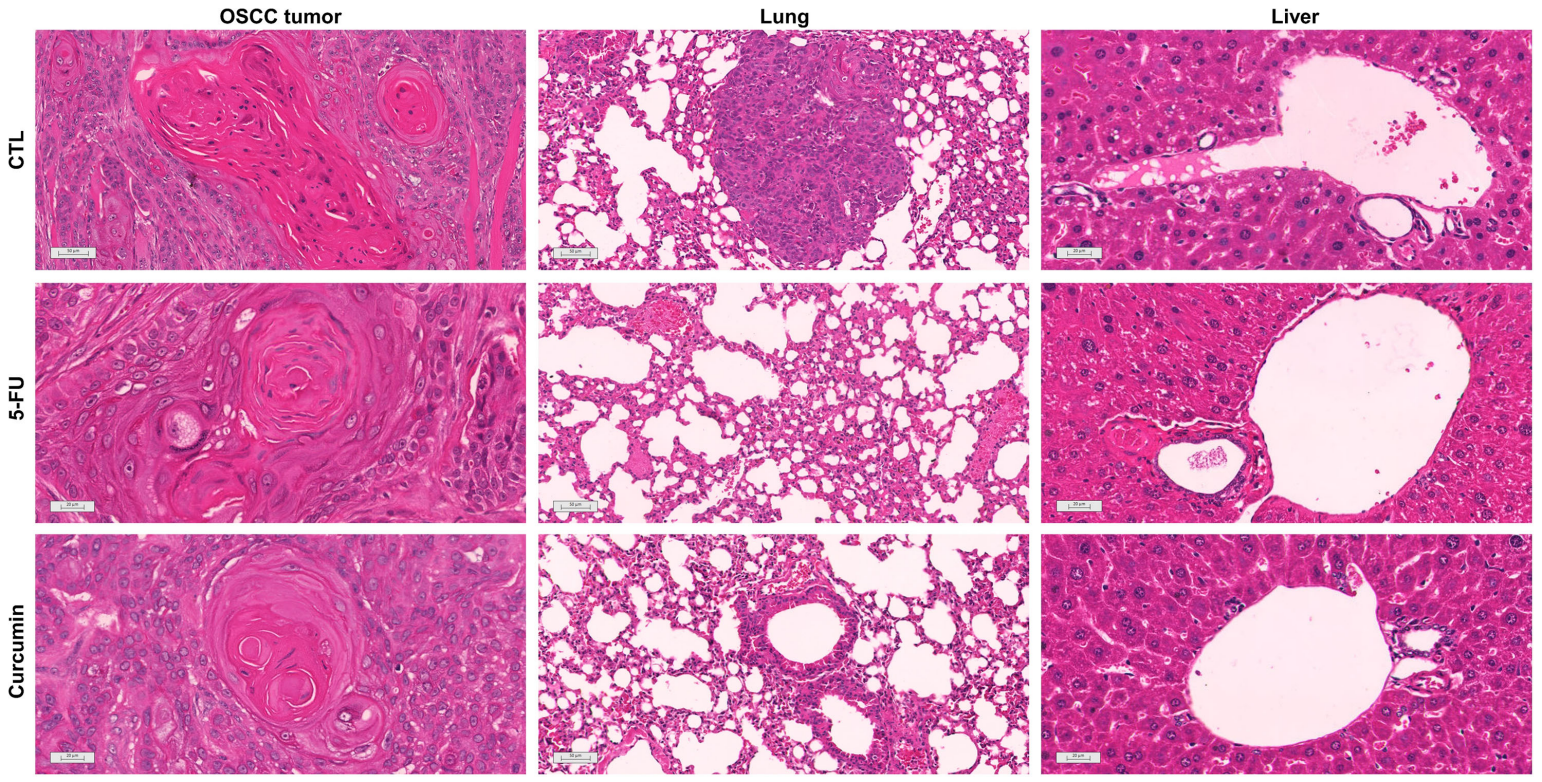
Representative photomicrographs of OSCC tumors, lungs, and livers of animals treated with curcumin. Histological sections were stained with hematoxylin-eosin and analyzed by light microscopy. A heterotopic xenograft model was employed to evaluate *in vivo* antitumor effects, with 24 mice receiving subcutaneous injections of HSC-3 cells (1 × 10^7^ cells/500 μL) in the left axilla. The negative control (CTL) was treated with the vehicle (5% DMSO) used for diluting the compounds tested, and 5-fluorouracil was used as a positive control.

Histological analysis showed that HSC3 tumors displayed features typical of OSCC, including marked cellular and nuclear pleomorphism, hyperchromatism, abnormal mitotic figures, hyperkeratosis, and squamous-like cells. Tumor grading ranged from moderately to well-differentiated in the negative control and 5-FU treatment groups, while tumors in the curcumin group were consistently well-differentiated. In all groups, tumor cells formed nodules or cords, surrounded by a poorly vascularized collagen matrix. Additionally, granulation tissue was present at the tumor edges, and areas of coagulative necrosis (comedonecrosis) were often seen, especially in the central tumor regions. Inflammatory infiltrates, mainly mononuclear cells, were primarily located next to necrotic areas. Invasion into nearby adipose tissue, muscle, and nerves was observed across all groups (**Figure 8**).

Histological examination of the organs showed that the heart and kidneys retained their tissue architecture (Data not shown). The lung parenchyma exhibited partial preservation with thickened alveolar septa and moderate atelectasis. Inflammatory infiltrates, characterized by a predominance of polymorphonuclear cells and intense vascular hyperemia, were observed across all experimental groups. Tumor nodules and emboli were particularly noted in the lungs of the negative control group (**Figure 8**). The liver showed partial preservation of architecture, with notable moderate vascular hyperemia and mild hydropic degeneration. Inflammatory cells, both polymorphonuclear and mononuclear, were found adjacent to hepatic sinusoids and portal vessels. These pathological changes were particularly pronounced in the livers of animals treated with curcumin and 5-FU.

The systemic toxic effects of curcumin were assessed after treatment. No significant differences were seen in body and organ wet weights (**Supplementary Figure S4**). Among the hematological parameters analyzed, such as erythrocytes, hemoglobin, and mean corpuscular volume (**Supplementary Figure S5**), no differences were observed, but animals treated with 5-FU (15 mg/kg) showed significant leukopenia and neutropenia compared to the negative control (**Supplementary Figure S6**).

## 4 Discussion

To contribute to the investigation of NC with potential for chemotherapy in OSCC, this study shows that curcumin exerts cytotoxic activity by inducing ROS-mediated apoptosis and effectively suppresses tumor growth *in vivo*. This investigation is the first to describe how curcumin promotes apoptosis induced by oxidative stress in metastatic OSCC, as well as its effects on cell viability in a 3D scaffold-free model and on tumor growth in an *in vivo* model.

First, the cytotoxicity of curcumin was tested in HSC3 cells cultured in a monolayer. This compound showed a significantly lower EC_50_ value compared to the positive control, reinforcing its therapeutic potential and suggesting the possibility of minimizing side effects. According to a prior study (10), a lower effective concentration of a compound enhances its therapeutic potential by reducing adverse effects associated with systemic drug administration. In addition, studies with other OSCC cell lines found higher concentrations of curcumin, including the YD10B, SCC-15, and Hep-2 cells that exhibited an IC_50_ of 10 µM (36, 40), and the H-314 and ORL-15 cell lines, where the IC_50_ was determined to be 50 µM (41).

Curcumin markedly reduced HSC3 cell numbers and induced concentration- and time-dependent morphological alterations, including cell shrinkage and enhanced granularity, consistent with apoptosis. These results are consistent with studies that demonstrated the potential of curcumin to induce apoptosis in OSCC cell lines (41–43). In the present study, treatment with curcumin resulted in an increased proportion of cells undergoing late apoptosis. These findings are consistent with previous studies (40, 41), which also reported enhanced apoptosis after 24 hours of exposure to curcumin.

Here, mitochondrial superoxide levels were measured in OSCC cells, revealing a significant increase after curcumin treatment. This finding is supported by studies that show curcumin is associated with ROS production (25, 40, 42, 44). Additionally, pretreatment with the antioxidant NAC reduced both early and late apoptosis in OSCC cells. Similar results were reported by Kim et al. (2012) (40), who observed that ROS production induced by curcumin (10 µM) in YD10B OSCC cells was nearly completely inhibited in the presence of NAC.

For the 3D model, a previously published protocol was applied to obtain a three-dimensional (3D) culture of OSCC, using a scaffold-free/magnetic technique (38). HSC3 cells were incubated with biocompatible NanoShuttle™ (magnetic nanoparticles) composed of iron oxide, gold, and poly-l-lysine. These nanoparticles do not promote any effect on cell morphology, viability, or function, even in activating important processes such as oxidative stress or inflammatory response, as previously described (45–47). Here, Curcumin exhibited greater toxicity in the 3D cell culture compared to 2D cell cultures, corroborating the greater therapeutic resistance described for this model (48, 49). According to Hoarau-Véchot (2018) (50), in the 3D model, cells are more resistant to the action of drugs because they present behavior closer to that observed *in vivo*. Therefore, due to the configuration and cellular interactions of the 3D system, drugs tend to present a higher effective concentration in this model when compared to the monolayer culture. Thus, spheroids treated with curcumin displayed cellular disaggregation, reflecting its cytotoxic effects, consistent with observations from other studies employing spheroid models for drug screening (51, 52).

Considering that 3D culture is an alternative but does not replace animal models (50, 53) and given the lack of *in vivo* studies using OSCC cells, the effect of curcumin was evaluated in an *in vivo* model. In this study, Curcumin (50 mg/kg) significantly reduced tumor growth, likely due to apoptosis, as we previously showed in monolayer culture. Prior studies (10, 13, 25) have also established this correlation in animals. Additionally, histological analysis showed variability in the grading of OSCC tumors within the same experimental group, supporting the findings of Kakhet et al. (2020) (25).

The antitumor potential of Curcumin has been applied in a range of studies, not only *in vitro* (41–43, 49) but also *in vivo* (10, 12, 25) and clinical trials (54, 55). There is no consensus on concentration limits for testing NC. However, the pharmaceutical industry commonly adopts EC_50_<10 μM, and concentrations above 30–50 μM are discouraged (16). In this way, many reported doses may not be pharmacologically meaningful, and the various activities described in the literature are dose and time-dependent. Moreover, it is important to monitor the activity of NC (extracts, fractions, purified compounds) through at least three purification steps in order to establish the correlation between chemical purity and biological activity (15). In addition, bioactivity claims for NC are meaningful when a clear relationship to the activity of isolated pure compounds is established, highlighting the need to correlate the observed effects with isolated NC to validate their bioactivity (56, 57). In the last years, bioavailability, effectiveness, side effects and patient trials undergoing chemo-radiotherapy have been developed and data obtained confirm that curcumin has potential in the treatment of cancer patients (25–27). Nevertheless, poor aqueous solubility, bioavailability, and pharmacokinetic profiles limit curcumin’s therapeutic usage. In order to improve its bioavailability, different formulation techniques have been investigated (10, 58, 59). Furthermore, well-controlled clinical trials that demonstrate efficacy, safety, optimal dosing, and pharmacokinetics in humans are still required to validate its therapeutic potential and to translate preclinical findings into clinical practice (60). Thus, the scarcity of *in vivo* studies with OSCC cells and the absence of clinical trials have restricted understanding of Curcumin’s role in oral cancer, and it remains uncertain whether long-term treatment would produce similar benefits.

These results provide evidence for the oxidative potential of Curcumin in metastatic oral cancer cell lines, while also underscoring the need for further research to confirm its role as an adjuvant in chemotherapy. Study limitations include the lack of assessment of curcumin in non-tumor cell lines (to calculate the selectivity index) and evaluation in only a single metastatic cell line, without including non-metastatic cells to better replicate the tumor microenvironment. Future studies are recommended to investigate the efficacy and bioavailability of different curcumin dosages and formulations, as well as potential synergistic effects with established chemotherapeutic agents. Ultimately, well-designed, large-scale clinical trials are essential to establish the safety, tolerability, and efficacy of curcumin alongside standard antineoplastic therapies, which may benefit patients undergoing treatment for various cancers, including OSCC, a disease characterized by significant resistance to conventional treatments, leading to high morbidity and mortality.

## 5 Conclusion

**Figure 9:**
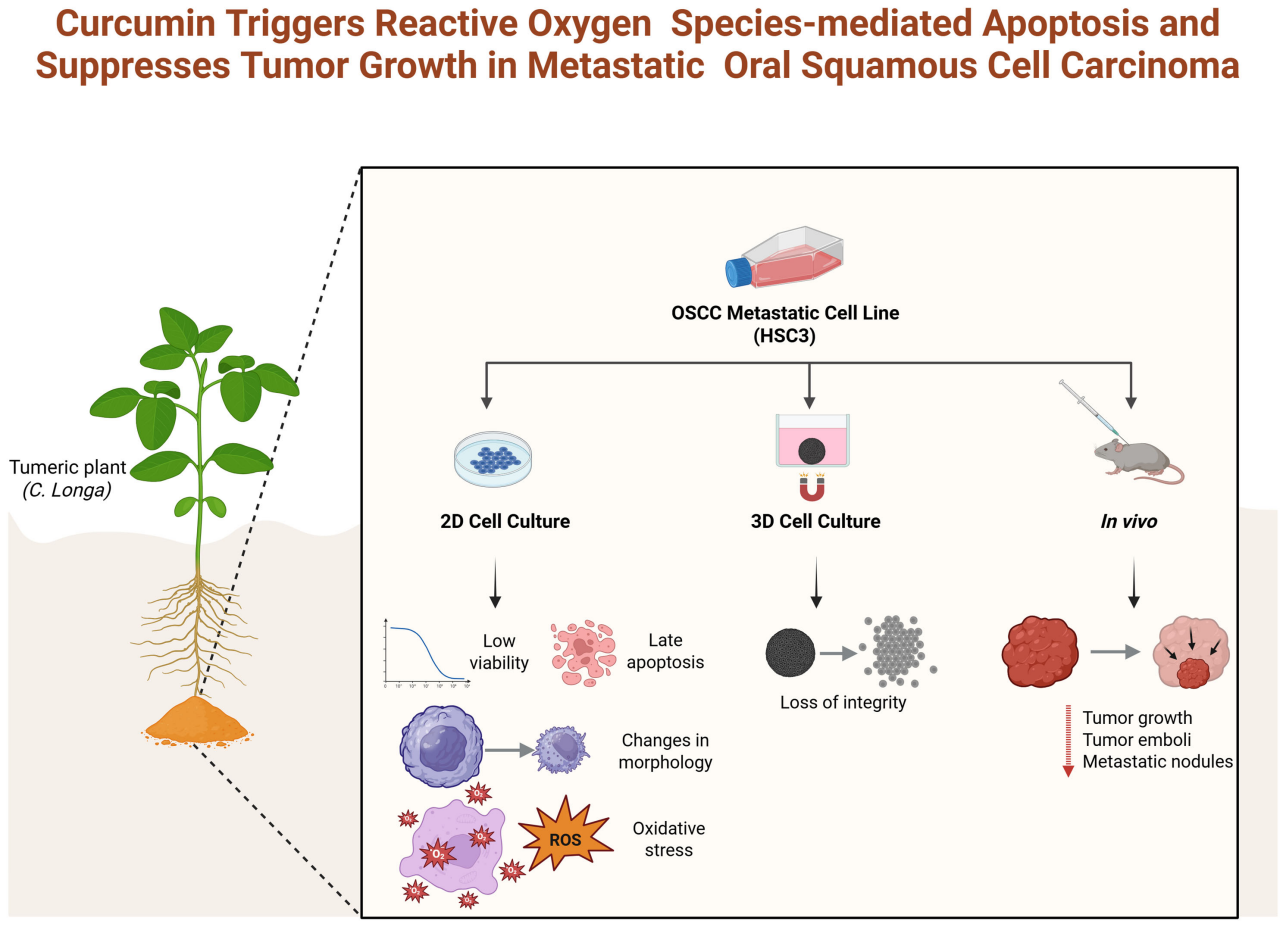
Overview of curcumin’s therapeutic effects *in vitro* and *in vivo* on HSC-3 cells. Created with BioRender.com..

Based on the results obtained, curcumin demonstrated promising cytotoxic activity against metastatic OSCC cells in both 2D and 3D models, with the effect being concentration- and time-dependent, and with tumor spheroids exhibiting greater resistance to treatment. Furthermore, this compound induced apoptosis mediated by increased reactive oxygen species (ROS) production and morphological alterations, as well as reduced tumor formation *in vivo* (**Figure 9**). Despite these encouraging findings, curcumin is not, to date, an FDA-approved drug for cancer treatment, and its clinical relevance remains uncertain. The mechanistic connection between curcumin and OSCC is not yet fully understood, and significant limitations such as its poor bioavailability, instability, and variability across formulations must be critically addressed. Therefore, although this study highlights the potential antitumor activity of curcumin in preclinical models, more rigorous pharmacological, toxicological, and clinical investigations are essential before considering curcumin as a viable therapeutic candidate for oral cancer.

## Data Availability

The original contributions presented in the study are included in the article/**Supplementary Material**. Further inquiries can be directed to the corresponding author.
