# Supplementary material for: Curcumin triggers reactive oxygen species-mediated apoptosis and suppresses tumor growth in metastatic oral squamous cell carcinoma
Source: Front Oncol. 2025 Oct 10;15:1668271. doi: 10.3389/fonc.2025.1668271 (PMC12549243; doi:10.3389/fonc.2025.1668271)
Supplement: Supplementary file 1 [file DataSheet1.pdf]

Supplementary Material

1 Supplementary Data

Supplementary Material should be uploaded separately on submission. Please include any supplementary data, figures and/or tables. Supplementary material is not typeset so please ensure that all information is clearly presented, the appropriate caption is included in the file and not in the manuscript, and that the style conforms to the rest of the article.

2 Supplementary Figures and Tables

2.1 Supplementary Figures

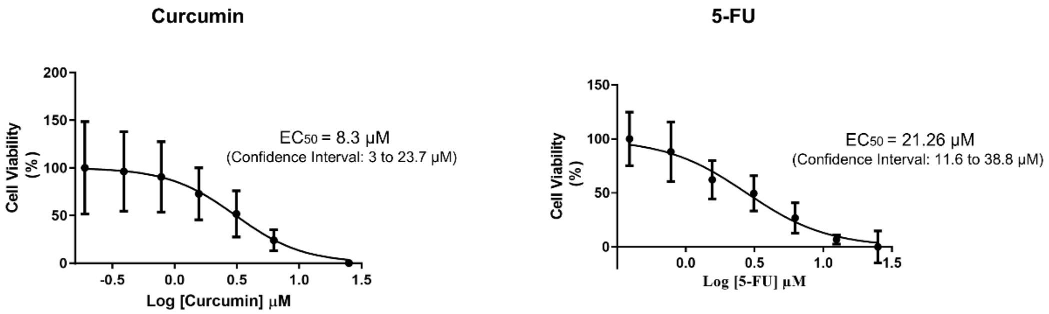

Supplementary Figure S1. Cytotoxicity curves of Curcumin and 5-FU in HSC3 cell line using the monolayer model. The data are presented as EC50 values corresponding to µM with a 95% confidence interval obtained by non-linear regression of three independent experiments carried out in triplicate. Cytotoxicity was evaluated using the CellTiter-Glo® reagent after 24 hours of incubation with the drugs. The 0.5% DMSO group was used as a calibrator.

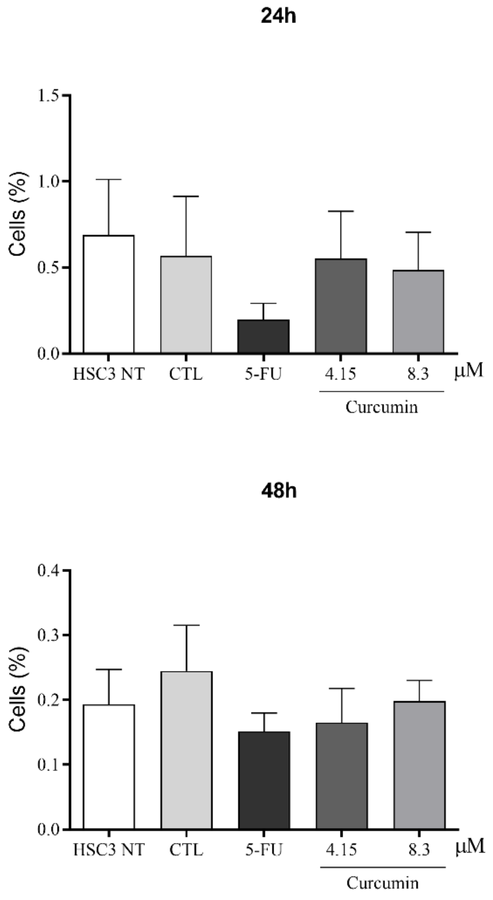

Supplementary Figure S2. Analysis of the population of necrotic cells after Curcumin treatment, in HSC3 cells, after 24 (A) and 48 (B) hours of treatment. The negative control was treated with the vehicle (DMSO) used to solubilize and dilute the substances, and 5-FU was used as a positive control. Data are representative of three independent experiments carried out in duplicate. Cellular debris was omitted from analysis, and 10,000 events were analyzed per sample. (*) $p \leq 0.05$ when compared to the negative control (DMSO) and (#) when compared to HSC3-NT (non-treated cells) by ANOVA (analysis of variance) followed by Student Newman-Keuls test.

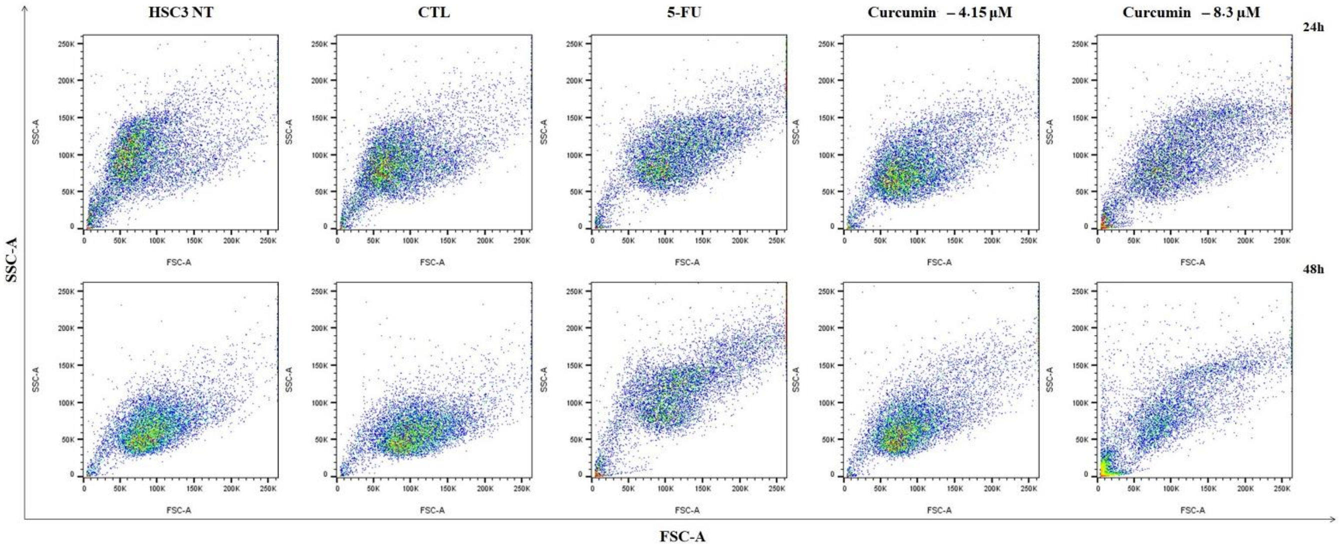

Supplementary Figure S3. Representative dot plots of light scattering in HSC3 cells treated with Curcumin. FSC (Forward scatter) and SSC (Side scatter), determined by cytometry, were used as parameters of relative size and granularity or internal complexity of the cell, respectively, after 24 and 48 hours of treatment. The negative control was treated with the vehicle (DMSO) used to solubilize and dilute the substances, and 5-FU was used as a positive control. Data are representative of three independent experiments carried out in duplicate. Cellular debris was omitted from analysis, and 10,000 events were analyzed per sample.

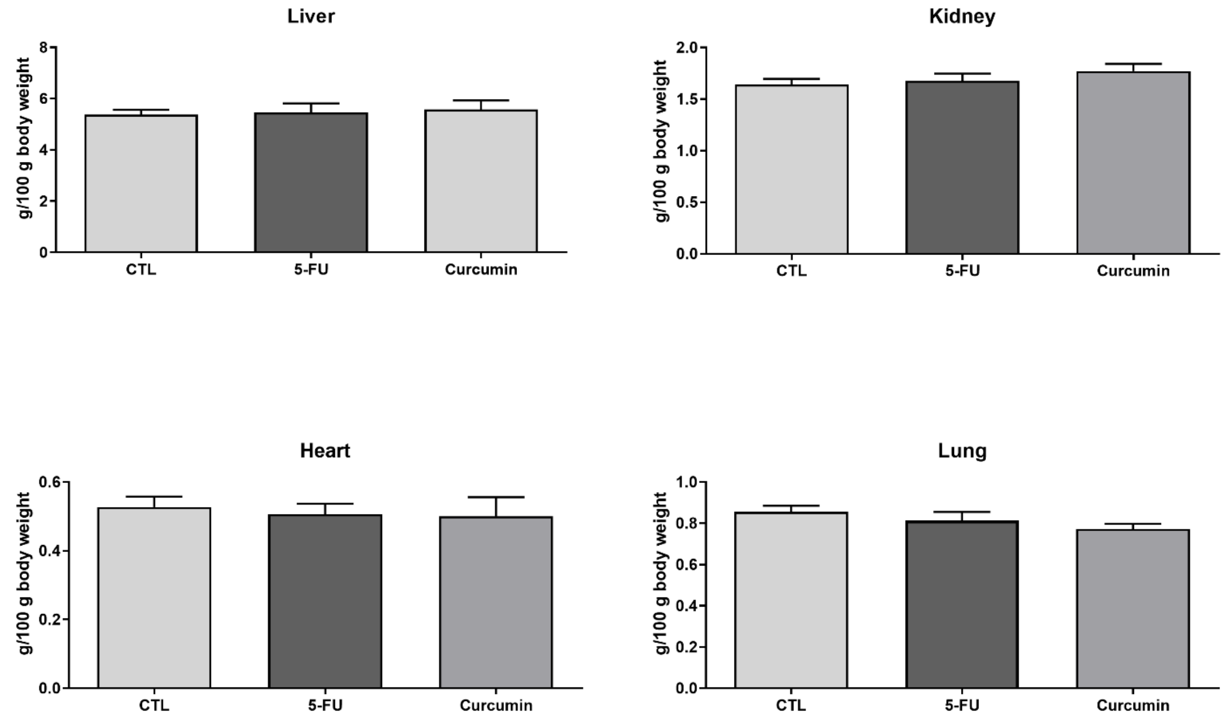

Supplementary Figure 4. Effects of curcumin on relative organ weight from C.B-17 SCID mice bearing HSC3 cells. The negative control group (CTL) received vehicle treatment (5% DMSO), while 5-fluorouracil (5-FU) was used as a positive control. Data are presented as the mean ± S.E.M. of three independent experiments performed in duplicate. * $p \leq 0.05$ compared with the negative control by ANOVA followed by Student Newman-Keuls test.

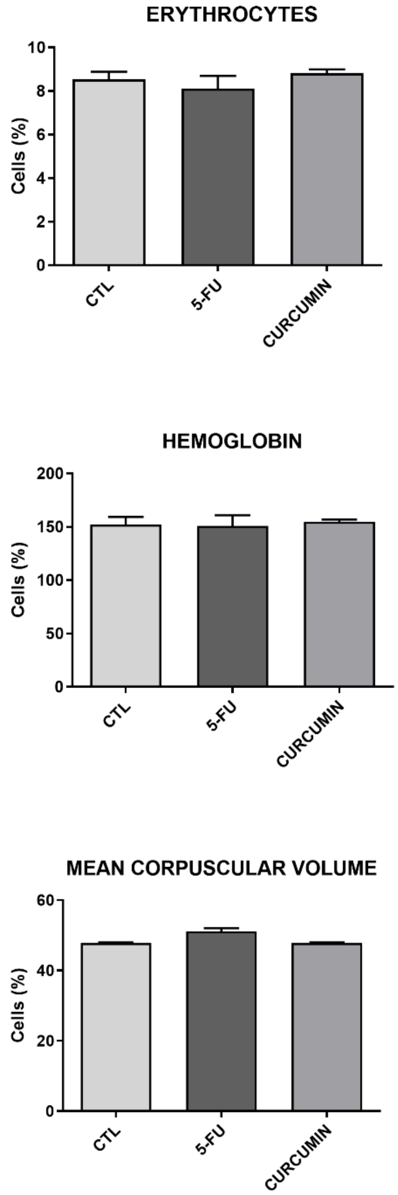

Supplementary Figure 5. Effects of curcumin on hematological parameters in the peripheral blood of C.B-17 SCID mice implanted with HSC-3 tumor cells. The negative control group (CTL) received the vehicle (5% DMSO), while 5-fluorouracil (5-FU) was used as a positive control.

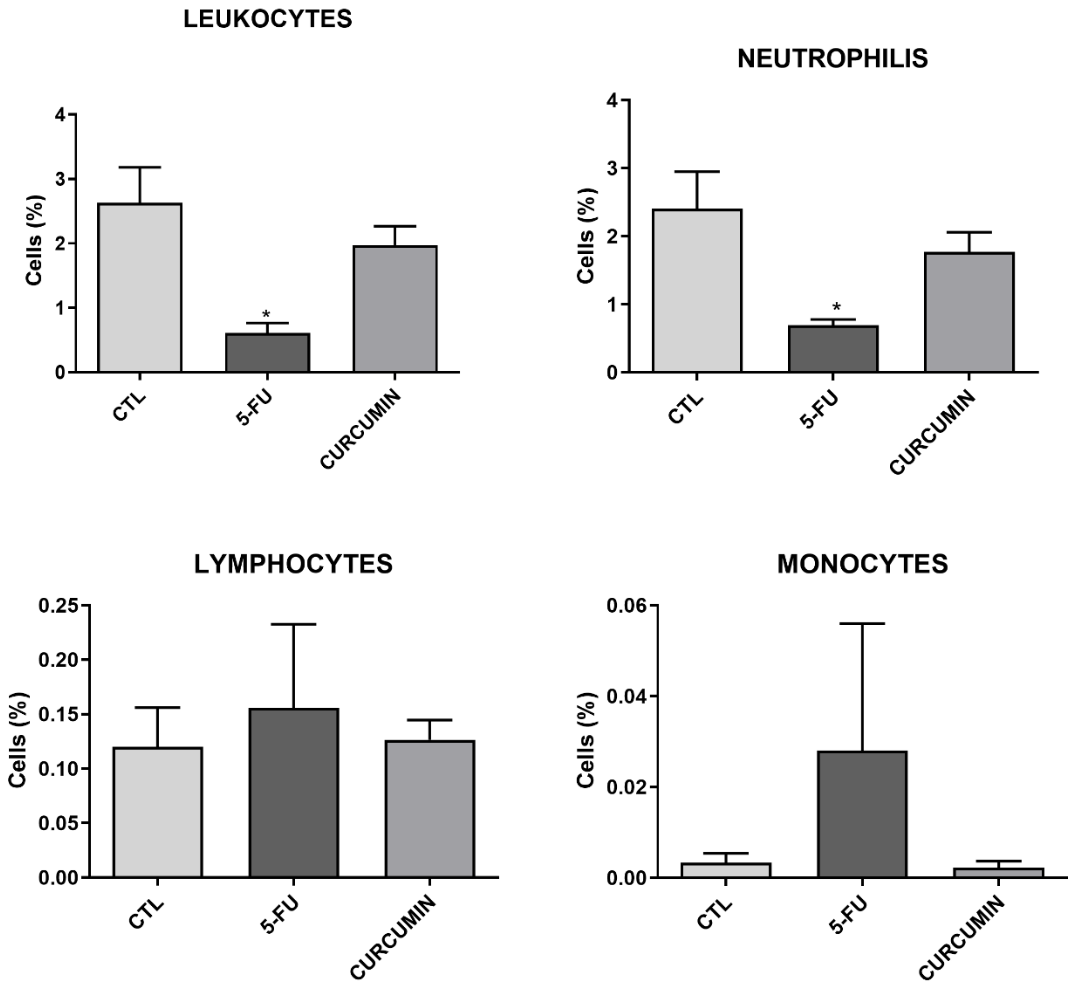

Supplementary Figure 6. Effects of curcumin on peripheral blood leukogram parameters in C.B-17 SCID mice bearing HSC-3 tumor cells. The negative control group (CTL) received vehicle treatment (5% DMSO), while 5-fluorouracil (5-FU) was used as a positive control. (*) $p \leq 0.05$ compared with the negative control by ANOVA, followed by the Student–Newma–Keuls test**.**
